# The Effect of Different Bleaching Protocols, Used with and without Sodium Ascorbate, on Bond Strength between Composite and Enamel

**DOI:** 10.3390/ma13122710

**Published:** 2020-06-15

**Authors:** Maroun Ghaleb, Giovanna Orsini, Angelo Putignano, Sarah Dabbagh, Georges Haber, Louis Hardan

**Affiliations:** 1 Department of Restorative Dentistry, Dental School, Saint Joseph University, Beirut 11072180, Lebanon; maroun.ghaleb@net.usj.edu.lb (M.G.); sarah.dabbagh@net.usj.edu.lb (S.D.); georges.haber@usj.edu.lb (G.H.); louis.hardan@usj.edu.lb (L.H.); 2 Department of Clinical Sciences and Stomatology, School of Medicine, Polytechnic University of Marche, Via Tronto 10, 60126 Ancona, Italy; a.putignano@staff.univpm.it

**Keywords:** hydrogen peroxide, carbamide peroxide, sodium ascorbate, antioxidant, microtensile bond strength

## Abstract

This in vitro study aims to evaluate whether a solution of 10% sodium ascorbate (SA) may exert a beneficial effect on the bonding of composite to enamel after using different bleaching agents and protocols. Microtensile bond strength (µTBS) was evaluated on 72 freshly extracted human central incisors, divided into eight experimental groups and one control group (total n = 9): Group 1 serves as control (nonbleached). Group 2 was bleached with 5% carbamide peroxide. Group 3 was bleached with 5% carbamide peroxide and then treated with 10% SA. Group 4 was bleached with 10% carbamide peroxide. Group 5 was bleached with 10% carbamide peroxide, then treated with 10% SA. Group 6 was bleached with 16% carbamide peroxide. Group 7 was bleached with 16% carbamide peroxide, then treated with 10% SA. Group 8 was bleached with 6% hydrogen peroxide. Group 9 was bleached with 6% hydrogen peroxide, then treated with 10% SA. All groups were restored immediately after the different treatments using a resin composite. The µTBS values were measured using a universal testing machine and statistical analysis was performed by means of normality and variance analyses, SIDAK test for univariate test and multiple comparisons, and Student test to compare µTBS values of each group with the control. The mean µTBS values in groups 2, 4, 6, 8 were significantly lower than controls. For groups 3, 5, 7, 9, subjected to antioxidant (10% SA) application, all µTBS values increased significantly. However, only for Groups 3 and 5 there was no significant difference with the control. Applying 10% SA for 10 min may improve the bond strength composite/bleached enamel just when whitening is performed with 5% and 10% carbamide peroxide.

## 1. Introduction

Aesthetic dentistry has evolved considerably in recent times. Discolored, damaged, or misaligned teeth are no longer tolerated by patients, who increasingly desire to have the perfect smile. The smile has become a symbol of beauty, health, and an indicator of one’s self-esteem [1]. Many studies have demonstrated that the color of the anterior teeth is one of the most important factors in determining whether patients are satisfied with their smile [2]. As a result, teeth whitening has increased in popularity, due to its efficiency, simplicity, safety, low cost and conservative approach to treat discolored teeth [3].

Vital tooth bleaching consists of three different methods: at-home bleaching, in- office bleaching and usage of over-the-counter products. The research for the ideal whitening agent began in the 1800s. Oxalic acid, chloric solution, hydrogen bioxide, potassium cyanide are a few of the agents that were previously utilized [4]. Nowadays, peroxide has become the most popular agent. Hydrogen peroxide (HP) is used at concentrations ranging from 5% to 40% while carbamide peroxide (CP) is used at concentrations between 10% to 35% [4].

Previous studies have shown that whitening agents adversely decrease the microtensile bond strength (µTBS) of composite to the enamel when bonding is performed immediately after the bleaching process [5,6,7]. It is reported that the decrease of the bond strength is related to the presence of residual oxygen in the interprismatic space, which prevents the adequate infiltration of the adhesive and its polymerization [8,9]. The general approach to solve this situation is to delay the restorative procedures from 24 h to 3 weeks. A waiting period may not always be possible for many reasons such as a lack of time, traveling commitments, personal occasion or aesthetics reasons due to the difference in color between the old restoration and the tooth [10].

Many techniques have therefore been proposed to overcome the decrease in the bond strength caused by the bleaching of enamel. Some authors proposed the removal of the superficial layer of enamel [11]. Other reports have suggested the use of adhesives containing organic solvents [12,13,14].

The use of antioxidants has been proven in many studies to be a safe and effective solution to increase the bond strength directly after bleaching, with no significant difference with the control group (without bleaching) [15]. Natural antioxidants, as well as synthetic phenolic antioxidants can effectively inhibit oxidation and have been successfully used to increase the bond strength of a composite resin to bleached enamel [15,16,17].

Sodium bicarbonate, rosemary extracts, pedicularis extracts, *Aloe vera*, pomegranate peel, grape seed extracts, green tea, extracts of pine bark, proanthocyanidins are all antioxidants already used to remove residual oxygen from dental tissue after whitening [15,18]. In addition, some vitamins like α-tocopherol (active component of the vitamin E complex) and sodium ascorbate (SA) or ascorbic acid (vitamin C) are known as a neutral, biocompatible and potent antioxidants, with the ability to reduce various oxidative compounds. Hydrogen peroxide or carbamide peroxide have been reported to induce a reduction in the bond strength of resin composite to both enamel and dentin, a situation that could be reverted with the use of the antioxidant SA [19,20]. Kimyai and Valizadeh evaluated the use of SA formulated as a hydrogel or solution and found no differences between the preparations, demonstrating that bond strengths were significantly increased following the application of SA [16]. In particular, SA when used as a 10% solution and application time of 10 min allows the free radicals of the adhesive to continue their polymerization without premature interruption by restoring the altered redox potential of the oxidized adhesive substrate, thus leading to improved bonding [21]. However, there are studies that assessed the effective improvement of enamel-composite bond strength values after vital bleaching, by comparing different percentages of CP and HP- based newly launched at-home whitening agents.

Therefore, the purpose of the current research was to conduct an in vitro comparative study to evaluate whether 10% solution of SA would have a beneficial effect on the enamel-resin composite bonding after using different whitening gels as 5%, 10% and 16% carbamide peroxide (PC) and 6% hydrogen peroxide (PH).

## 2. Materials and Methods

**Figure 1:**
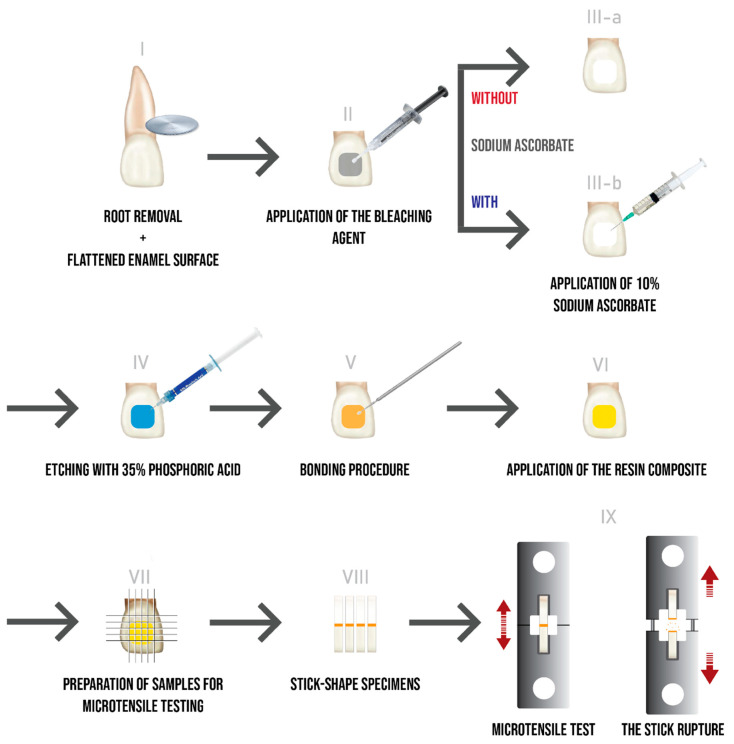
Schematic diagram illustrating the experimental procedures.

Seventy-two human recently extracted central incisors were collected and stored in a solution of 0.1% thymol. The protocol was approved by Ethical Committee of the Saint Joseph University (Beirut, Lebanon; ref.# USJ-2016-110) and all participants have signed informed consent before the extractions. The criteria for tooth selection were intact vestibular enamel, no fissures or cracks, no restorations and no caries. After extraction, the teeth were cleaned of any residual tissue tags with ultrasound, pumiced, washed under running tap water and prepared, as shown in Figure 1. Roots were cut 2 mm apically to the cementoenamel junction with a diamond disc. The vestibular surfaces were flattened using abrasive discs of aluminium oxide (Soflex 3M ESPE, St. Paul, MN, USA) with running water.

The teeth were randomly divided into one control group (n = 8) and eight experimental groups (n = 8): Group G1 is the control group, in which teeth were restored without any bleaching. Groups G2, G4, G6 and G8 were restored immediately after bleaching using 5% PC, 10% PC, 16% PC and 6% PH, respectively. In groups G3, G5, G7 and G9, following bleaching with 5% PC, 10% PC, 16% PC and 6% PH respectively, 10% SA solution was applied to the enamel surface for 10 min, and then teeth were restored.

### 2.1. Bleaching Procedure

All the teeth were bleached using “White dental beauty” bleaching material (Optident, Ilkley, UK). Four different concentrations of gel were used: 5% PC, 10% PC, 16% PC and 6% PH. The vestibular surface of the teeth was coated with the bleaching gel and kept for eight hours per day, for seven consecutive days. After daily application, teeth were thoroughly rinsed with water and air-dried for 30 s. For the remainder of the day, they were preserved in distilled water at room temperature. Visual color changes were observed after this procedure.

### 2.2. Application of Antioxidant

A 10% solution of SA was prepared by dissolving 10 g of ascorbic acid powder (L (+) ascorbic acid sodium salt, Sigma-Aldrich, St. Louis, MO, USA) in 100 mL of distilled water. Groups G3, G5, G7 and G9 were treated with this solution as follows: 10 mL of 10% SA solution (pH = 7.4) was applied onto the vestibular surface with a syringe, with a flow rate of 1 mL per minute, for 10 min in order to keep the surface wet. The teeth were then rinsed with distilled water for 30 S and dried using compressed air for 5 S to obtain a mat surface, prior to the bonding procedure.

### 2.3. Bonding Procedure

The vestibular enamel was etched with 35% phosphoric acid (3M/ESPE) for 30 s, then rinsed thoroughly for 30 s with distilled water. Afterwards, the bonding system (Single Bond 2, 3M/ESPE) was applied with a microbrush in two consecutive layers with 5 s air spray after each to evaporate the solvent, then cured for 10 s using Elipar Deep-cure-S (3M/ESPE).

A resin composite (Filtek Z250, 3M/ESPE) was incrementally placed directly on the enamel surface and cured for 20 s for each layer. The total height of the composite was measured using a periodontal probe, obtaining a total height of 4 mm. Then, the specimens were stored in distilled water at 37 °C for 24 h.

### 2.4. Specimen Preparation for µTBS Test

The teeth were embedded in transparent acrylic resin (Supacryl Ortho, Faprodent, Marrakech, Morocco) in a mold to facilitate the cutting procedure. Afterwards, these samples were cut using a cutting machine (Exakt Technologies Inc., Norderstedt, Germany) to obtain sticks, formed by 4 mm of resin composite and 4 mm of tooth structure. (Figure 1) The sticks had a size of 1 × 1 × 8 mm^3^ and were selected according to the following criteria: all samples had to be 8 mm long and extrathin (<0.8 mm) or extra thick (>1.2 mm) sticks were excluded; they were confirmed to be defect-free under a light microscope (thus not presenting voids, irregularities due to the cutting process and irregularities) [18].

In order to assess the µTBS, each stick was individually fixed to a special device with flowable composite (Filtek™ Supreme Ultra Flowable Restorative, 3M ESPE). After that, it was adapted to the YL-01 (YLE GmbH, Bad König, Germany) testing machine at a crosshead speed of 1 mm/min.

The µTBS test was run until the sticks were ruptured. The obtained value (in Newtons) was then recorded (see Figure 1, IX), then divided by the adhesion area (in mm^2^) to convert it to mega Pascal (MPa). A digital caliper (Mitutoyo, Tokyo, Japan) was used to measure the adhesion area.

Two or three sticks from each tooth were taken, and µTBS values recorded, measuring them in MPa and calculating the mean for each tooth.

### 2.5. Statistical Analysis

Statistical Package Software for Social Sciences (SPSS for Windows, SPSS Inc., Chicago, IL, USA, version 24.0) was used for statistical analysis of data. The level of significance was *p*-value ≤ 0.05. Kolmogorov-Smirnov tests were performed to assess the normality of the distribution of quantitative variables.

Two-factor variance analyses were conducted to compare the µTBS between the different bleaching systems based on the presence or absence of SA. These tests were followed by univariate analyses and multiple comparisons of SIDAK.

Student tests were conducted to compare the mean µTBS at the level of each group with the control group.

## 3. Results

### 3.1. Mean µTBS Composite/Enamel Values after Using Different Bleaching Products

Mean and standard deviation of the µTBS values of composite to enamel in teeth treated using different bleaching products, based on the presence or absence of SA are illustrated in Table 1.

#### 3.1.1. Comparison between the Presence or Absence of Sodium Ascorbate (SA)

This study showed that the presence of SA significantly increases the mean µTBS of composite to enamel in teeth treated with 5% PC (*p*-value < 0.001), 10% PC (*p*-value < 0.001), 16% PC (*p*-value < 0.001) and 6% PH (*p*-value < 0.001) (Table 1).

#### 3.1.2. Comparison among Bleaching Products

In the presence of SA, the µTBS was significantly higher in group G3 (5% PC) (*p*-value < 0.001) than in other groups; the difference was not significant among the groups G5 (10% PC), G7 (16% PC) and G9 (6% PH) (*p*-value = 1.000).

**Figure 2:**
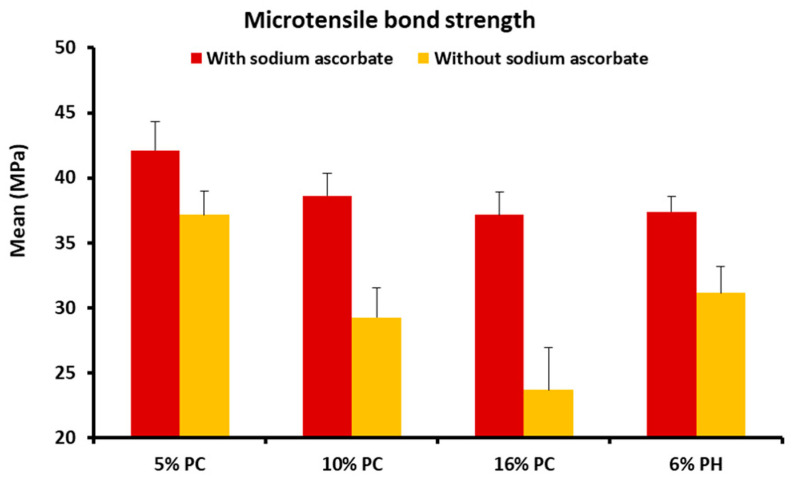
Mean microtensile bond strength (μTBS) values of each group. (PC: carbamide peroxide; PH: hydrogen peroxide).

In the absence of SA, the highest µTBS was found in group G2 (5% PC) (*p*-value < 0.001), followed by the groups G4 and G8 (10% PC and 6% PH) (*p*-value < 0.001), whereas lower values were found in group G6 (16% PC) (*p*-value < 0.001); note that the difference was not significant between the groups G4 and G8 (*p*-value = 0.477), as shown in Figure 2.

### 3.2. µTBS Values in Different Bleaching Products and Control Group

The mean and standard deviation of the µTBS values for the different bleaching products based on the presence or absence of SA, is shown in Table 2. The µTBS of the control group G1 was not significantly different when considering groups G3 (5% PC with SA) (*p*-value = 0.277) and group G4 (10% PC without SA) (*p*-value = 0.097); by contrast, it was significant if considering groups G2 (5% PC without SA) (*p*-value = 0.006), G4 (10% PC without SA) (*p*-value < 0.001), G6 (16% PC without SA) (*p*-value < 0.001), G7 (16% PC with SA) (*p*-value = 0.006), G8 (6% PH without SA) (*p*-value = 0.006), as shown in Table 2.

## 4. Discussion

Microtensile bond strength test is the most common test used for assessing the bond strength of dental materials [5,6,7]. In this study, it has been used to evaluate the neutralizing effect of 10% SA solution on the µTBS of a composite to enamel, immediately after bleaching, using different whitening systems.

Many authors have studied the bonding strength directly after whitening. The majority concluded that a significant reduction in bond strength compared to control groups (without whitening) occurs [5,6,7]. The results of this study are in agreement with the previous results, showing a significant reduction of the bond strength values of the four bleaching products concentrations (5%, 10%, 16% PC and 6% PH) compared to the group of unbleached enamel.

Our results are in agreement with those of Topcu et al. who showed that the enamel-resin bond strength is significantly decreased when the restoration is performed directly after bleaching with the 10% and 16% PC concentrations [22].

In addition, even with the use of a low concentration of 5% PC, a significant reduction in bonding strength compared to the control group has occurred. The same result was established by Cura et al., who used low concentrations of 3% and 10% PC and noted that even with these concentrations, the bond strength is significantly reduced [23].

In the present study, the results show that the higher the PC concentration, the lower the immediate bond strength, as proven also by Türkün et al. in 2004 [24].

There are several reasons for reducing the bond strength directly after whitening. The main cause of this reduction is the presence of residual oxygen in the enamel and dentin which prevents the infiltration of the adhesive into the etched enamel or partially inhibits its polymerization. Oxygen is a highly reactive chemical element. It will react with the free radicals of the adhesive, inhibit their polymerization and generate polymers with reduced mechanical properties [8,9].

Other studies have explained that this reduction in bond strength is due to the demineralization of dental tissues, the reduction of their surface energy and the denaturation of dentinal collagen following bleaching. These structural changes are directly proportional to the concentration and time of application of the whitening product [25].

Titley et al. microscopically observed the morphological alteration of the hybrid layer and resin tags after whitening with 35% hydrogen peroxide. They concluded that enamel thinning, large surfaces are free of adhesive, resin tags are less numerous, short, fragmented and not well defined [26].

Sodium ascorbate, the sodium salt form of ascorbic acid, is known as a neutral, biocompatible and potent antioxidant with the ability to reduce various oxidative compounds such as oxygen [21]. Since ascorbic acid has a very low pH of 1.8, its use is not recommended in clinical procedures to avoid damaging the dental tissues. In contrast, SA, which has the same antioxidant activity as ascorbic acid with higher pH = 7.4, makes it more compatible for use on dental tissues. For this reason, SA has been chosen as an antioxidant product instead of ascorbic acid [27].

Regarding the application time and concentration of SA, in our study a 10-min period and a concentration of 10% have been adopted, consistent with several studies using low concentrations of PC and PH [27,28,29]. This combination of application time and concentration has been shown to be effective at reversing the reduced bonding strength after whitening. Thapa et al. have demonstrated that after whitening with 10% carbamide peroxide, the 10-min application of 10% or 25% SA significantly improves the µTBS between enamel and composite resin [30].

The results of this study demonstrated that the bonding strength is significantly improved following the application of SA for 10 min, directly after bleaching, using the four concentrations of whitening products. These findings are in agreement with other studies, which explain that the bond strength is improved due to the fact that SA allows the free radicals of the adhesive to continue their polymerization without premature interruption by restoring the altered redox potential of the oxide adhesive substrate [14].

However, in groups G7 (16% PC with SA) and G9 (6% PH with SA), even though the values are close to the control group, this improvement is insufficient to be statistically significant. Theoretically, a solution of 16% PC decomposes into 5.35% PH and 10.65% urea [4]. According to this logical reasoning, the results should therefore be similar, for these two concentrations.

Many studies note a significant increase in bond strength values between composite resin and enamel whitened with different bleaching concentrations and treated with 10% SA [29,31,32].

Sasaki et al. compared two antioxidants after whitening with 10% PC: 10% SA and 10% α-tocopherol in both solution and hydrogel forms. They evaluated the shear bond strength of human dentin and enamel submitted to bleaching treatment with 10% carbamide peroxide, then treated with antioxidant agents containing 10% SA and 10% α-tocopherol formulated in solution and gel, concluding that treatment with 10% α-tocopherol solution was the most effective way to reverse the bond strength on the bleached enamel [20].

Ozelin et al. compared 10% SA with green tea, evaluating their potential to reverse the reduction of bond strength after application for 15, 30 and 60 min on the enamel bleached with 10% carbamide peroxide. They established that the bond strength values in both antioxidants-treated samples were higher than the value in the bleached group, only when they were applied for 60 min [33]. On the other hand, another report demonstrated that the application of 10% SA for 10 min was sufficient to reverse the decrease in bond strength, but the SA was used in solution form and the enamel samples were continuously agitated using a sterile brush [24]. A possible explanation of these divergent results may be due to the different antioxidant forms (e.g., gel or solution) and the methodology used for the evaluation (e.g., microshear or microtensile bond strength test) [33].

In addition, after usage of 10% SA for 10 min on enamel bleached with 15% carbamide peroxide, Sharafeddin et al. noticed a significant improvement in the bond strength compared to the control group (unbleached enamel). However, they claimed that the effect of antioxidant on bond strength decreases with the decrease of the concentration of the bleaching agent [34].

In our protocol a 10% solution of SA has been used, which, according to Kimiyai et al., showed no significant difference in bond strength with respect to the use of the two forms, solution and hydrogel, of SA [16]. On the other hand, the improvement in bond strength obtained in our study by a 10-min SA application is in partial disagreement with their findings, showing that a treatment of 10% SA for a duration of 10 min is not sufficient to reverse the reduced bond strength following bleaching with a low concentration PC of 10%, while a duration of three hours improves this bond strength to insignificant results compared to the control group (unbleached enamel) [16].

Alencar et al. concluded that there is no statistical difference between the unbleached group (control) and the bleached group (treated with 10% SA for 15 min). These values remain reduced compared to those found in groups with a waiting time of 7 days [35].

Noteworthy is that in group G3 (5% PC with 10% SA) the bond strength increased insignificantly compared to the control group, when 10% SA was applied for 10 min (G3 = 42.09 MPa and G1 = 40.7 MPa). Similar results were obtained by Vidhya et al., who compared the effect of SA and grape seed extracts on bond strength. The difference was significant for groups treated with grape seed extracts only [36].

The present research represents the first report comparing the effect of different concentrations of bleaching agents, used with and without SA, on the µTBS between composite and enamel. It would be stimulating, in the future, to illustrate these findings by means of scanning electron microscopy, and to better explore the possible chemical mechanism leading to SA mode of action by X-ray spectroscopy [18,37]. One limitation of this study consists on the fact that SA effect was studied only on enamel, whereas it would have been interesting to explore its activity also on dentin, in case of non-vital bleaching, which will be, indeed, a possible aim of our further study [19,20]. Moreover, it could be necessary to perform similar bleaching protocols for a period longer than one week, in order to mimic other commonly used patient’s bleaching treatments, which may last up to 2 or 3 weeks. Additional studies will be also needed to significantly improve the µTBS to the bleached enamel (16% PC or 6% PH), by changing the mode of application of SA. This can be evaluated by increasing the concentration or the duration of SA application.

## 5. Conclusions

In summary it can be concluded that:

(1) Bond strength values were found to be the lowest, when the restorative procedure was carried out immediately after bleaching with 5%, 10%, 16% carbamide peroxide and 6% hydrogen peroxide.
(2) The higher the bleaching product concentration is, the lower the immediate bond strength.
(3) The application of 10% SA for a duration of 10 min significantly increases the bond strength.
(4) When SA was applied following bleaching with 5% and 10% CP only, there was no significant difference in the µTBS values compared to the control group.

Treatment of the bleached teeth with 10% SA before bonding can be very useful for clinicians as it reverses the compromised enamel-composite bond strength. Caution should be considered when using 6% hydrogen peroxide or 16% carbamide peroxide.

## Figures and Tables

**Table 1:** Mean microtensile bond strength in different groups (PC: carbamide peroxide; PH: hydrogen peroxide; N: number of specimens). Different letters indicate the presence of significant difference; same letters indicate no significant difference.

Groups	With Sodium Ascorbate	Without Sodium Ascorbate	*p*-Value
5% PC	42.10 ± 2.194 ^b^ (N = 8)	37.17 ± 1.792 ^c^ (N = 8)	<0.001
10% PC	38.62 ± 1.736 ^a^ (N = 8)	29.25 ± 2.271 ^b^ (N = 8)	<0.001
16% PC	37.16 ± 1.725 ^a^ (N = 8)	23.68 ± 3.253 ^b^ (N = 8)	<0.001
6% PH	37.40 ± 1.182 ^a^ (N = 8)	31.13 ± 2.062 ^b^ (N = 8)	<0.001
*p*-value	<0.001	<0.001	

**Table 2:** Comparison of the microtensile bond strength of every group with the control group. (PC: carbamide peroxide; SA: sodium ascorbate; PH: hydrogen peroxide).

Groups	Bleaching Treatments	Difference of Means	*p*-Value	95% Confidence Interval	
				Lower	Upper
Control	5% PC without SA	3.5600	0.006	1.0668	6.0532
	5% PC with SA	−1.3675	0.277	−3.8607	1.1257
	10% PC without SA	11.4800	0.000	8.9868	13.9732
	10% PC with SA	2.1050	0.097	−0.3882	4.5982
	16% PC without SA	17.0513	0.000	14.5580	19.5445
	16% PC with SA	3.5700	0.006	1.0768	6.0632
	6% PH without SA	9.6013	0.000	7.1080	12.0945
	6% PH with SA	3.3288	0.010	0.8355	5.8220
